# Inventory of Obsolete Pesticide Warehouses in Tajikistan and Implications for Removal of Contaminated Soil

**DOI:** 10.5696/2156-9614-8.17.1

**Published:** 2018-03-12

**Authors:** Umidjon Amonovich Ulugov, Lyudmila Sergeevna Bobritskaya, Julia Sinitsky

**Affiliations:** 1. Pure Earth, New York, USA; 2. Peshsaf, Tajikistan

## Abstract

**Background.:**

Tajikistan is an agrarian-industrial republic. A large portion of the Tajikistan economy relies on agriculture. With the rise of agriculture, especially cotton production, came the widespread use of pesticides. Abandoned and unsupervised pesticide storage warehouses have become a health and environmental problem. In many cases such sites, as well as significant areas of pesticide-contaminated land, remain accessible to the public. A survey and analysis of 26 former pesticide storage warehouse sites across Tajikistan revealed a country-wide pesticide exposure problem that threatens the health of vulnerable populations. Infrastructure and local governance issues are barriers to addressing this health crisis for rural residents.

**Objectives.:**

The purpose of this article is to describe the issues surrounding former pesticide storage warehouses and their effect on the health of the population of Tajikistan.

**Materials and Methods.:**

In 2012, the non-governmental organization (NGO), Pure Earth (formerly Blacksmith Institute), with the financial support of the Food and Agriculture Organization of the United Nations (FAO), Green Cross Switzerland and the European Union conducted surveys of 26 pesticide warehouses located throughout Tajikistan. The survey included detailed site assessments, including analysis of the maintenance of chemicals and soil testing.

**Results.:**

Soil samples taken from the studied sites exceeded maximum permissible concentrations referenced by Pure Earth by several hundred times for dichlorodiphenyl-trichloroethane (DDT) and hexachlorocyclohexane (HCH), as well as aldrin and dieldrin. Even the most polluted sites had families living on the premises.

**Conclusions.:**

Pesticide contamination in Tajikistan is a serious and ongoing problem that requires the attention of local, state governments, and international non-governmental organizations. We recommend the development of a government-sponsored public education campaign to inform the population about the potential risks of exposure to obsolete pesticides. The dangers of agricultural use, former storage warehouses and disposal areas should be addressed. In addition, data from the 2012 surveys of 26 warehouses should be used to prioritize the four high-risk sites and develop preliminary clean-up plans for contaminated soil around warehouses to present to government ministries and NGOs.

**Competing Interests.:**

The authors declare no competing financial interests

## Introduction

Tajikistan is an agrarian-industrial republic. The area of cultivated crops is about 830,000 hectares.^1^ Agriculture accounted for 29.2% of Tajikistan's gross domestic product in 2015.^2^ The development of agriculture, especially cotton production, in Tajikistan is closely related to the widespread use of pesticides to combat agricultural pests, plant diseases and weeds. The agroclimatic conditions of the republic are very favorable for the rapid reproduction of many agricultural pests. In addition, there is a high prevalence of plants with various diseases.

A great deal of attention was paid to the agricultural sector in the republic when it was part of the former Union of Soviet Socialist Republic (USSR). In the Tajik Soviet Socialist Republic, there were significant areas of agricultural land for growing crops, especially cotton. Millions of rubles were spent to build infrastructure and to provide the agriculture industry with agrochemicals and pesticides. Once the Soviet Union collapsed, so did the complex infrastructure of pesticide monitoring and warehouse maintenance.

At present, independent Tajikistan is facing a number of unresolved issues. Among these issues are abandoned and unsupervised pesticide storage warehouses, which are in many cases accessible to the public, as well as significant areas of pesticide-contaminated land adjacent to these structures, also open to public access. Lack of oversight from the transitioning government in the 1990s and early 2000s led to these and a number of other unsafe practices regarding former pesticide storage sites.

According to the World Health Organization (WHO) in Tajikistan, the mean concentration of dichlorodiphenyl-trichloroethane (DDT) in human breast milk is 4 times higher than the biomonitoring equivalent permissible level of 2,300 ng/g lipids.^3^ The high content of DDT in breast milk may be attributable to the population coming into contact with DDT through unauthorized and illegal dumping of pesticides near living quarters, as well as the continued use of available stocks of banned pesticides in agriculture, especially in household plots.

According to the National Action Plan (NIP) for the implementation of the obligations of the Stockholm Convention on persistent organic pollutants (POPs) in the Republic of Tajikistan, a survey of 167 warehouses with a total content of about 240 tons of pesticides was conducted prior to 2007.^4^ These warehouses are subject to legal challenges with regard to ownership between the Ministry of Agriculture and local executive bodies. With the abolition of the Republican Research and Production Association (which operated in the Soviet period), most of these warehouses and surrounding land were privatized. Their condition, control over use, cleaning and procedures for issuing construction permits on privatized areas have come under the control of local authorities. The following summarizes the survey results:

- All standard warehouses of the former Republican Research and Production Association were privatized, and some were completely destroyed, and the building material used for the construction of houses and other facilities.
- Almost all existing warehouses of former collective and state farms are in a dilapidated state, many of them without windows and doors, and many have faulty roofs.
- Warehouses and adjacent areas are significantly contaminated with pesticides.
- Often, warehouses do not have fencing and protection, allowing access by the local population and animals.
- Some warehouses are located near populated areas and buildings, close to water sources, crops and fruit plantations.
- Some warehouses are used as storage for animal feed, various building materials and household implements.
- There are cases of warehouses being used as dwellings for families with small children despite an acrid smell permeating the properties.

## Objectives

Prior to 2012, the government of Tajikistan had surveyed pesticide warehouses, but detailed studies to determine the impact on public health, especially the concentration of chemicals in the soil, were not conducted. The purpose of this article is to present results of a follow up survey conducted from 2012 to 2017. The follow up survey highlighted issues surrounding former pesticide storage warehouses and related effects on the health of local populations. It also addresses the negative consequences of changing the legal status of warehouses without regard to the health outcomes that result from these changes.

**Abbreviations**
*DDT*: Dichlorodiphenyl-trichloroethane
*HCH*: Hexachlorocyclohexane

## Materials and Methods

In 2012, Pure Earth, with the financial support of the United Nations Food and Agriculture Organization of the United Nations (FAO), Green Cross Switzerland and the European Union, conducted surveys of 26 of the 372 total pesticide warehouses located throughout Tajikistan. The 26 pesticide warehouses were selected by Pure Earth from 132 warehouses in Tajikistan identified as posing the greatest risk to public health by the Committee for Environmental Protection under the Government of the Republic of Tajikistan. The 26 warehouses selected for the follow up survey were the ones to which Pure Earth had the best access.

The follow up survey included detailed site assessments using a well-established protocol as well as information collected through discussions with local people and officials. The follow up survey was conducted as part of the Pure Earth Toxic Sites Identification Program (TSIP).^5^

The 26 warehouses were assessed for the maintenance of chemicals, including their concentrations, over the last 5 years. The follow up survey was, first of all, aimed at identifying sources, migration routes, and receptors, and employed risk-based approaches to prioritize sites where people are most likely to be affected and involved preliminary sampling and analysis. The level of sampling and analysis performed was not, however, detailed enough to develop a detailed cleanup plan, but did assist in prioritization of sites for remediation.

Soil analysis was performed in specialized laboratories in Tajikistan (Institute of Chemistry under the Academy of Science of the Republic of Tajikistan) and Kyrgyzstan (LLC Scientific - Production Association “ILIM”). The laboratories remain from the Soviet period.

## Results

The surveys found that most of the warehouses of the former Republican Research and Production Association (RRPA – under the Ministry of Agriculture) had been privatized by a variety of local actors and in many cases have been almost completely destroyed. Residential houses have been built near the warehouses, and there are personal plots on which fodder, vegetables and fruit crops are grown. In a number of cases, people have settled in buildings belonging to these former warehouses.

Analyses of soil samples taken from the studied sites showed that all sites exceeded either the United States Environmental Protection Agency (USEPA) or Canadian Environmental Quality Soil Guidelines for DDT and hexachlorocyclohexane (HCH), as well as aldrin and dieldrin^6^,^7^

Out of the 26 sites assessed, Djami was the most contaminated, according to the lab data. There was a strong scent at the site, and people nearby reported difficulty breathing and headaches. Local residents excavated the site and uncovered 400 cubic meters of contaminated soil, sacks of pesticides and iron barrels containing unknown substances. In the same area, in Djovidon Jamoat, according to laboratory data, maximum concentrations for HCH (all forms) was more than 800 ppm, and in the Jaihun area (formerly Kumsangir), the maximum concentration for DDT exceeded 1000 ppm. These sites all had families living on the premises. All sites exceeded USEPA and Canadian guidelines.^6^,^7^

The four most contaminated sites, ranked according to the Blacksmith Index,^8^ are included in Table 1.

**Table 1:** Most Contaminated Obsolete Warehouses Surveyed

**TSIP Database Number**	**Site Name**	**Blacksmith Institute Index (maximum of 10)**	**Key Pollutant**	**Key Pollutant Concentration mg/kg (in soil)**	**Recommended Allowable Level mg/kg (in soil)^6^,^7^**	**Estimated Population Affected**
TJ-3208	Pesticide Burial Site, Village 1, Kumsangir Region	7	DDT	1,074	1.7	2,000
TJ-4989	10 years of independence, Khamadoni area	5	HCH (all forms)	18.89	0.52	319
TJ-4958	Djovidon, F. Saidov, Djami district	5	HCH (all forms)	82.51	0.52	50
TJ-4992	Ittifoq, Khamadoni area	5	DDT	30.074	1.7	1,163

**Abbreviations**: TSIP, Toxic Sites Identification Program

## Conclusions

The follow up survey results found that the local population is able to purchase land contaminated by pesticides and other chemicals. Many people were left with contaminated plots in the distribution of land during the post-communist transition.

In many areas, pesticides remain stockpiled or are illegally imported. Unfortunately, these illegal supplies are not always identified and detected by the government. In addition, unauthorized and illegal dumping of pesticides continues.

The level of sampling and analysis performed was not detailed enough to develop a detailed cleanup plan, but did assist in prioritization of sites for remediation.

Of great concern for researchers is the possible illegal and unauthorized burial of pesticides in land surrounding the former warehouses, which are currently used by the local population which privatized them. This creates ethical conflicts where researchers and local officials may be forced to relocate entire families to pesticide-free areas, temporarily depriving them of homes.

The consequences of privatization, as well as the use of formerly restricted safety zones around former warehouses for housing construction, negatively affect the health of the local population, especially women and children. According to the WHO, on average, DDT levels in breast milk in Tajik women are 4 times greater than the biomonitoring equivalent permissible level of 2,300 ng/g lipids.^3^ This is despite the fact that there has been a ban on the use of DDT in agriculture in the former USSR since 1970, and a ban on using DDT to combat malaria since 1985. This indicates that in Tajikistan, the use of this insecticide most likely continues. It is likely that local residents use expired batches for private use.

We believe that pesticide contamination in Tajikistan is a serious and ongoing problem that requires the attention of local and state governments, as well as international non-governmental organizations.

Beyond providing laboratories, the government of Tajikistan does not have the financial means to resolve these problems, and appropriate infrastructure is lacking. State structures do not have sufficient expertise, technical or institutional capacity for an independent study of the remaining facilities (according to the National Center for Implementation of the Stockholm Convention on Persistent Organic Pollutants, there may be up to 170 such warehouses), or for cleaning of contaminated areas and removal of contaminated soil and pesticide residues.

Tajikistan is in need of significant international assistance to clean up contaminated sites from obsolete pesticides.

We highly recommend the following steps to resolve this issue:

1. Improve existing legal and institutional frameworks in the Republic of Tajikistan to protect the rights and legitimate interests of citizens affected by pesticide-contaminated territories.
2. Develop a government-sponsored public education campaign to inform the population about the potential risks of exposure to obsolete pesticides through agricultural use or from former storage warehouses or disposal areas.
3. Using data from the 2012 surveys of 26 warehouses, prioritize the four high-risk sites and develop preliminary clean-up plans for contaminated soil around warehouses to present to government ministries and non-governmental organizations (NGOs).
4. To evaluate clean-up technology, attract specialists from the Emergency Situations Committee (labor force), the Ministry of Health and Social Protection of Population (for repeat analyses of persistent organic pollutants (POPs) content in breast milk), and the Committee for Environmental Protection under the Government of the Republic of Tajikistan (to assist with cleaning). The efforts of NGOs and experts can be used to conduct additional assessments that will be needed to develop detailed plans and methods for appropriate for clean-up of the land.
5. The Vakhsh polygon (a site for temporary storage of contaminated soil in Tajikistan) may be an acceptable disposal site for contaminated soil from the warehouses. Before considering use of the polygon as an acceptable disposal location, it should first be evaluated for suitability by technical experts. To be sustainable and environmentally protective, the land disposal site must be located in an area where it poses a low risk to groundwater, it should have an impermeable and nondegradable liner and appropriate cap, and it should have physical and legal controls to prevent access and improper use under current and future conditions.
6. The cost of this work will depend on the size of contaminated sites and the need for additional work, for example, dismantling of the walls of the former warehouses, their removal to the landfill for further burial, or runoff control to prevent the spillage of channels from the contaminated site. On average, according to our estimates, up to 25,000 USD may be required (for example, Saidov, Djami); large sites (for example, Village No. 1, Kumsangir district) may require 200,000 USD or more.
7. Provide support to the local population which may lose housing due to clean-up measures.
